# Speech analysis for differentiating bipolar disorder and major depressive disorder during euthymic states

**DOI:** 10.1186/s12991-026-00652-7

**Published:** 2026-04-02

**Authors:** Jhen-Wu Lai, Ya-Han Hu, Ya-Mei Bai, Cheng-Che Shen

**Affiliations:** 1 Department of Psychiatry, Taipei Veterans General Hospital, Taipei, Taiwan; 2 School of Medicine, National Yang-Ming Chiao-Tung University, Taipei, Taiwan; 3 Department of Information Management, National Central University, Taoyuan; City, Taiwan; 4 Asian Institute for Impact Measurement and Management, National Central University, Taoyuan City, Taiwan; 5 Jianan Psychiatric Center, Tainan, Taiwan

**Keywords:** Bipolar disorder, Major depressive disorder, Euthymic state, Speech analysis, Machine learning, Vocal biomarkers

## Abstract

**Objective:**

Differentiating major depressive disorder (MDD) from bipolar disorder (BP) is crucial for early diagnosis and targeted treatment. This study investigates speech, linguistic biomarkers, and machine learning models to differentiate between the MDD and BP patients using speech analysis during symptom-free periods.

**Methods:**

Voice recordings from 191 patients (41 BP, 150 MDD) during euthymic phases were analyzed. Linguistic metrics, sentiment scores, and acoustic features were extracted and compared between the groups. Various machine learning models were employed to classify diagnoses based on these features.

**Results:**

Significant differences were observed between BP and MDD patients. BP patients exhibited a shorter pause durations per total speaking duration and total word count (0.19 vs. 0.29; *p* < 0.05, 0.07 vs. 0.12; *p* < 0.05). MDD patients showed greater variability in speech intensity and peak amplitude during euthymic states. Sentiment analysis revealed no significant differences in broader emotional dimensions. Among the models tested, the Random Forest classifier achieved the highest predictive performance (AUC = 0.807), underscoring its effectiveness in differentiating BP from MDD.

**Conclusion:**

Speech analysis during euthymic periods reveals distinct linguistic and acoustic patterns between MDD and BP patients. These findings highlight the potential of speech features as non-invasive biomarkers for mood disorder classification, offering promising avenues for enhancing clinical decision-making. However, given the sample size, the results are preliminary and warrant replication in larger, independent cohorts.

**Supplementary Information:**

The online version contains supplementary material available at 10.1186/s12991-026-00652-7.

## Introduction

Differentiating between the major depressive episodes (MDEs) of major depressive disorder (MDD) and bipolar disorder (BP) is crucial for accurate diagnosis, treatment, and patient outcomes [1]. When a BP patient is assessed in a depressive phase, there is a relative risk of more than 40% of being mistakenly diagnosed as MDD [2]. The prediction of which patients will become bipolar after an index diagnosis of MDD is of considerable clinical importance.

Studies examining the spoken language in affective disorders date back as early as 1938 [3]. Voice analysis is emerging as a promising biomarker for detecting mood episodes in BP and MDD patients. Studies have confirmed that, compared to healthy controls (HC), individuals in the depressive phase exhibit distinct linguistic characteristics and can serve as a tool for predicting the onset of depression [4–13]. Commonly observed vocal characteristics in MDEs include reduced pitch variability, slower speaking rate, lower volume, and increased pauses or speech latency [14, 15]. Studies using acoustic analysis tools have found that MDD patients in depressive states frequently exhibit decreased fundamental frequency and amplitude, suggesting lower pitch and diminished vocal intensity [16, 17]. Additionally, changes in prosody, or the rhythm and stress patterns in speech, contribute to a “flat” vocal effect in depressive patients [12]. As for manic phase detection, a study by Kaczmarek-Majer et al. used a smartphone app to monitor BP patients over time, finding distinct voice patterns that correlated with affective states, with male patients displaying increased loudness and pitch in manic phases, while females exhibited quieter, lower-pitched tones during similar phases [18]. Another study in South Korea, using wearable devices and smartphones to predict mood episode recurrences in patients with MDD, bipolar I disorder (BP I), and bipolar II disorder (BPII), achieved accuracies of 90.1%, 92.6%, and 93.0% for impending (mood swings occurred in 3 days) major depressive, manic, and hypomanic episodes, respectively [19].

Various studies using machine learning models have demonstrated that voice features significantly differ between individuals with different mood states [16, 20–22]. One study applied machine learning to distinguish adults with BP via daily phone data collection; they found that voice features were more accurate than automatically generated objective smartphone data on behavioral activities [20]. The area under the curve (AUC) in the classification of manic or mixed states was 0.89 compared with an AUC = 0.78 for the classification of depressive states [20]. Another study collected naturalistic phone calls made by the participants to distinguish between MDD and BP patients, the classification model achieved an AUC of 0.58 (SD: 0.07) [21]. Additionally, prosodic and spectral features derived from daily phone calls also serve as valid markers for the severity of depressive and manic symptoms in BP, allowing for phase prediction with accuracies exceeding 70% [18]. A systemic review focused on the studies on the smartphone-based systems that monitor or detect the phase change in BP reported accuracies in the range of 67% to 97% in predicting mood status (euthymic, depressive, manic, and mixed states) [22].

While previous studies have demonstrated that voice features can serve as reliable biomarkers for distinguishing manic and depressive episodes from euthymic states, limited research has explored differentiating between BP and MDD specifically during euthymic periods. To the best of our knowledge, only one prior study has utilized naturalistic phone calls to differentiate between MDD and BP during euthymia, achieving a modest AUC of 0.43 (SD: 0.16) [21].

In this study, we apply data mining and speech analysis techniques to examine the spoken narratives of individuals diagnosed with MDD and BP during their euthymic phases. The primary objective of this study is to leverage voice data obtained during symptom-free periods to differentiate between BP and MDD.

## Methods

### Data sources and study sample

We compiled a Chinese speech data related to chief complaints from 191 individuals (150 with MDD, 41 with BP) at a northern Taiwan medical center. All participants were ≥ 18 years old, with no history of traumatic brain injury or schizophrenia. Each case included demographic data, audio recordings, and scores from the Hamilton Depression Rating Scale 17 (HAMD-17) [23], Young Mania Rating Scale (YMRS) [24], and Clinical Global Impression (CGI) [25].

To ensure a euthymic state, inclusion criteria required HAMD-17 ≤ 7, YMRS ≤ 12, and CGI ≤ 2. Audio recordings were collected by asking: “Please describe your recent emotions, life circumstances, and other states within one minute.” in a controlled environment.

### Ethics statement

The study was approved by the Institutional Review Board (IRB) of Taichung Veterans General Hospital (IRB number: SE22066A). All participants provided written informed consent after receiving details on the study’s purpose, procedures, potential risks and benefits, confidentiality measures, and their voluntary participation rights.

*Research* Variables.

Clinical diagnoses (MDD or BP) were the dependent variable in the predictive models. We identified 118 independent variables, categorized into three groups. Demographic variables [10] covered participant background. Textual variables (102) included 13 from Part-of-Speech (POS) analysis, 75 from term frequency-inverse document frequency (TF-IDF), and 14 from sentiment analysis. Audio variables [6] were derived from audio analysis.

### Text preprocessing

Our study used Google’s Cloud Speech-to-Text API to transcribe audio, providing automatic segmentation, speaker identification, and punctuation insertion. We calculated Total Word Count and derived Speaking Rate by dividing word count by speech duration. Speaking Rate, classified as a textual variable, excludes acoustic features.

For Part-of-Speech (POS) analysis, we used Python’s jieba (posseg) package, identifying Adjectives, Conjunctions, Adverbs, Numerals, Nouns, Verbs, Measure Words, Prepositions, Pronouns (First, Second, Third Person), Time, Particles, and Negation terms. Negation variables were counted using a custom dictionary (e.g., not, no). We also used NLTK’s Chinese stop word list to calculate Stop Words as an independent variable.

After the stop word count, we removed these words from the text and applied the TF-IDF method using the *Scikit-learn* package in Python3 to extract feature word vectors ranging from 50 to 500 in size. TF-IDF is a common weighting technique used in information retrieval and text mining to evaluate the importance of a word within a specific document relative to a collection of documents [26]. Term Frequency (TF) measures how frequently a word appears in a given document, while Inverse Document Frequency (IDF) assesses the rarity of a word across all documents in the dataset. The core principle of TF-IDF is to highlight words that are frequent in a specific document but rare in others, as these words are more likely to carry meaningful information for distinguishing between categories [27]. The TF-IDF score for each word is calculated using the following formula:

1 $$\:{TF-IDF}_{i,\:j}=\:{TF}_{i,\:j}\:\times\:\:{IDF}_{i}$$

where *i* represents the index of the extracted term (ranging from 50 to 500), and *j* refers to the text from the *j*-th patient.

For each text, we computed TF-IDF scores to quantify the significance of individual words. From these results, we identified 75 keywords **(**e.g., *work*, *sleep*, *doctor*, *body*, *stress*) as independent variables. Furthermore, we calculated two additional metrics: the TF-IDF Top Word Sum, which represents the cumulative TF-IDF score of the extracted keywords, and the TF-IDF Top Word Sum/Word Count, which is the average TF-IDF score per word in each text.

### Sentiment analysis

Following text pre-processing, the refined terms were incorporated into a two-part sentiment analysis. Firstly, we utilized the Augmented National Taiwan University Sentiment Dictionary (ANTUSD) [27] to assess the polarity of the textual sentences. ANTUSD is widely used in Chinese sentiment analysis, offering nuanced interpretations of Chinese text [28, 29]. Using the CopeOpi system, we generated polarity scores ranging from − 1 to 1, accounting for negation terms preceding target words [28]. The polarity score for each word was calculated using the following formulas:

2 $$\:Score\left(i\right)=Negation\times\:Orientation\left(w\right)$$

3 $$\:Negation\:=\:\left\{\begin{array}{c}1\:\:if\:no\:negation\:exists\\\:-1\:\:if\:negation\:exists\end{array}\right.$$

4 $$\:Orientation\:\left(w\right)\:=\:\left\{\begin{array}{c}>0\:\:if\:positive\\\:<0\:\:if\:negative\end{array}\right.$$

The aggregated polarity scores across all words in a text were used to derive the CopeOpi Numerical Sentiment Score variable. Additionally, we computed the frequencies of Positive, Neutral, Negative, Non-opinionated, and Not-a-word annotations, normalizing these frequencies by the total word count to create corresponding numerical variables.

Secondly, we extracted eight emotional variables, including Anger, Anticipation, Disgust, Fear, Joy, Sadness, Surprise, and Trust, using the NRC Emotion Lexicon [29], a widely recognized benchmark for textual emotion detection [30–32]. The frequency of words associated with each emotion was normalized by the total word count. Similar to ANTUSD, negation terms were handled using a weighted multiplication approach, following Formulas [2] to [4].

### Audio analysis

For voice analysis, we used Cool Edit Pro 2.1, first applying Noise Reduction to minimize background noise, then Delete Silence function to remove pause intervals. In accordance with conventions in speech and psycholinguistic research, the term “pause” is used in this study to distinguish meaningful interruptions in speech from brief silences that naturally occur between words or syllables. Pause detection parameters were set at 35 dB, with a minimum duration of 200 ms and a maximum duration of 500 ms. This threshold range was selected because pauses shorter than approximately 150 to 200 ms are generally considered articulatory gaps rather than cognitively mediated pauses. Although pause thresholds vary across studies, we did not explore alternative cutoffs to maintain methodological consistency and avoid introducing additional variability into the pause-derived measures. The cumulative pause duration was calculated by subtracting the modified audio length from the original. From this, we derived two key variables:

- Pause Duration per Total Speaking Duration – calculated by dividing the total sum of pauses between sentences by the total speaking time (in seconds).
- Pause Duration per Total Word Count – calculated by dividing the total sum of pauses between sentences by the total number of words spoken.

Further, variables related to peak amplitude, including Minimum Peak Amplitude, Maximum Peak Amplitude, and Average Peak Amplitude, were automatically extracted using the Statistics Analyze feature. The Average Peak Amplitude was calculated by multiplying the Average Root Mean Squared (RMS) Power by 1.414, as peak amplitude is 1.414 times greater than RMS.

### Descriptive statistics

We compared the distributions of the demographic variables, POS frequency variables, sentiment analysis variables, and audio variables between the MDD and BP groups by using independent t-tests and chi-squared tests. Due to the large number of comparisons across the various feature sets (Demographic, Linguistic, and Acoustic), we implemented the Benjamini-Hochberg (BH) procedure to control the False Discovery Rate (FDR). All reported *p*-values for group comparisons were adjusted using the BH-FDR method, and a corrected *p*-value of less than 0.05 was considered statistically significant.

### Prediction model development and evaluation

To classify MDD and BP cases, eight machine learning techniques were employed and compared: Decision Trees (DT) [33–35], K-Nearest Neighbors (KNN) [36], Light Gradient Boosting Machine (LightGBM) [37], Logistic Regression (LR) [35, 38], Support Vector Machines (SVM) [34, 39], Naive Bayes (NB) [40], Random Forest (RF) [35, 41, 42], and Extreme Gradient Boosting (XGBoost) [42, 43]. These methods have demonstrated strong performance in prior studies involving text and audio features for depression detection [44–46].

The dataset was split in a 4:1 ratio, with four-fifths allocated for training and one-fifth for testing. To address the issue of class imbalance in the training set, the Synthetic Minority Over-sampling Technique (SMOTE) was employed to balance the class distribution and enhance model learning. A 10-fold cross-validation approach was adopted to evaluate model performance during training. All models were developed and evaluated using the Scikit-learn library in Python (v3.7.13), with default parameter settings applied throughout.

All 118 identified independent variables (10 demographic variables, 102 textual variables, and 6 audio variables.) were included as input features for every machine learning classification model tested. In this study, we employed a machine learning classification framework rather than traditional statistical modeling (like regression, where covariates are explicitly controlled). Therefore, variables such as age, sex, and psychiatric hospitalization history were not included as traditional statistical covariates to be “corrected” for. Instead, the 10 demographic variables (which function as common covariates in traditional statistics) were treated as additional input features within the overall set of 118 features. This allowed the machine learning algorithms to internally assess the predictive utility and importance of demographic data alongside the speech and linguistic biomarkers.

To assess the performance of our classification models, we utilized standard evaluation metrics, including the area under the ROC curve (AUC), accuracy, F1 score, precision, and recall. These metrics were calculated based on the confusion matrix, with AUC serving as the primary indicator of overall model performance. In cases where differences in AUC between models were minimal, additional metrics such as accuracy, F1 score, and recall were considered to provide a more comprehensive evaluation.

## Results

Of the 191 patients included in the analysis, 41 were diagnosed with BP and 150 with MDD. The demographic comparisons between patients with BP and MDD are listed in Table 1. A significant difference between the two groups was in psychiatric hospitalization history (*p* < 0.05).

**Table 1:** Demographic Data of Patients with bipolar disorder (BP) and major depressive disorder (MDD)

Variable name	BP *N* = 41 (%)	MDD *N* = 150 (%)	Corrected *P* values
Sex			0.86
Male	12 (29.27)	38 (25.33)	
Female	29 (70.73)	112 (74.67)	
Age (years) ^a^	39.90 (15.16)	42.48 (17.39)	0.77
Education Level			0.77
Elementary school	0 (0.00)	9 (6.00)	
Junior high school	5 (12.2)	13 (8.67)	
Senior high school	15 (36.59)	50 (33.33)	
College degree or higher	21 (51.22)	78 (52.0)	
Occupation	24 (58.54)	73 (48.67)	0.77
Marriage			0.78
Unmarried	22 (53.66)	73 (48.67)	
Married	14 (34.15)	65 (43.33)	
Divorced	5 (12.2)	12 (8.0)	
Suicide History	17 (41.46)	52 (34.67)	0.77
Psychiatric Hospitalization History			< 0.05*
No psychiatric hospitalization history	20 (48.78)	112 (74.67)	
Hospitalized within the past year	2 (4.88)	17 (11.33)	
Hospitalized more than one year ago	19 (46.34)	21 (14.0)	
Residential Status			0.77
Living alone	6 (14.63)	31 (20.67)	
Living with family or friends	35 (85.37)	119 (79.33)	
Alcohol Consumption Pattern			0.77
Less than three times per week	38 (92.68)	144 (96.0)	
Three times per week and above	3 (7.32)	6 (4.0)	
Number of Physical Illnesses	0.68 (0.87)	0.42 (0.73)	0.26

^a^Median (interquartile range);

*Statistical significance

### Part-of-speech frequency of patients with BP and MDD

The linguistic data from BP and MDD were compared, focusing on several language-related variables, including the usage of various parts of speech and the frequency of first/second/third-person pronouns listed in Table 2. For most categories of word usage, no significant differences were found. The frequency of first/second/third-person pronouns was similar in both groups, with no significant differences.

**Table 2:** Part-of-Speech Frequency of Patients with bipolar disorder (BP) and major depressive disorder (MDD)

Variable name	BP *N*= 41^a^	MDD *N*= 150^a^	Corrected *P* values
Adjectives	0.0323 (0.0311)	0.0326 (0.0319)	0.99
Conjunctions	0.0031 (0.0020)	0.0028 (0.0033)	0.91
Adverbs	0.2204 (0.0754)	0.2173 (0.0829)	0.91
Nouns	0.1274 (0.0439)	0.1204 (0.0447)	0.77
Verbs	0.0551 (0.0366)	0.0646 (0.0399)	0.36
Measure Words	0.0269 (0.0163)	0.0286 (0.0237)	0.86
Prepositions	0.0324 (0.0239)	0.0315 (0.0226)	0.91
Pronouns	0.0324 (0.0239)	0.0315 (0.0226)	0.91
Time	0.0440 (0.0379)	0.0438 (0.0365)	0.99
Negations	0.0147 (0.0143)	0.0185 (0.0207)	0.68
First-Person Pronouns	0.0008 (0.0)	0.0026 (0.0)	0.63
Second-Person Pronouns	0.0310 (0.0382)	0.0442 (0.0536)	0.26
Third-Person Pronouns	0.0051 (0.0072)	0.0109 (0.0097)	0.41

^a^Mean (interquartile range);

*Statistical significance

### Sentiment analysis of patients with BP and MDD

The sentiment analysis of BP and MDD groups during euthymic status is listed in Table 3. The Copeopi Numerical Sentiment Score was mildly higher in BP patients (0.0302) compared to MDD patients (0.0250) and did not reach a statistical difference with a p-value of 0.05. No significant differences were observed in other sentiment dimensions and emotion-related sentiment variables.

**Table 3:** Sentiment Analysis of Patients with bipolar disorder (BP) and major depressive disorder (MDD)

Variable name	BP *N*= 41^a^	MDD *N*= 150^a^	Corrected *P* values
Copeopi Numerical Sentiment Score	0.0302 (0.0139)	0.0250 (0.0180)	0.25
Positive Annotation	0.1124 (0.0441)	0.1059 (0.0628)	0.77
Neutral Annotation	0.0116 (0.0168)	0.0127 (0.0169)	0.91
Negative Annotation	0.0636 (0.0507)	0.0696 (0.0453)	0.77
Non-Opinionated Annotation	0.0041 (0.0058)	0.0078 (0.0102)	0.51
Not-a-word Annotation	0.0003 (0.0)	0.0003 (0.0)	0.99
Anger	0.0133 (0.0186)	0.0160 (0.0220)	0.77
Anticipation	0.0313 (0.0254)	0.0334 (0.0235)	0.86
Disgust	0.0110 (0.0122)	0.0110 (0.0166)	0.99
Fear	0.0228 (0.0221)	0.0214 (0.0257)	0.90
Joy	0.0280 (0.0150)	0.0284 (0.0256)	0.99
Sadness	0.0209 (0.0207)	0.0217 (0.0227)	0.92
Surprise	0.0188 (0.0185)	0.0198 (0.0216)	0.91
Trust	0.0351 (0.0168)	0.0375 (0.0256)	0.83

^a^Mean (interquartile range);

*Statistical significance

### Audio analysis of patients with BP and MDD

Significant differences were observed across all variables in audio features comparisons except speaking rate (Table 4). The BP group had a higher total word count documented within the one-minute recording, however, did not reach statistical significance (201.1 vs. 146.2; *P = 0.08)*. The speaking rate was slightly higher for BP compared to MDD patients, but was not statistically significant (*P =* 0.44). The MDD group tended to pause more frequently and for longer durations during their speech (*P < 0.05*). Similarly, pause duration per total word count was lower in the BP group compared to the MDD group (*P < 0.05*). Peak amplitude was also significantly reduced in BP patients (*P < 0.05*), with similar trends for maximum and minimum peak amplitudes (*P < 0.05*). The average peak amplitude was reduced in BP compared to MDD (*P < 0.05*), underscoring that MDD patients tend to speak with greater variability in volume, speech intensity, and a generally higher peak amplitude compared to BP.

**Table 4:** Audio Analysis of Patients with bipolar disorder (BP) and major depressive disorder (MDD)

Variable name	BP *N*= 41^a^	MDD *N*= 150^a^	Corrected *P* values
Total Word Count	201.1463 (146)	146.2100 (101.5)	0.08
Speaking Rate	1.9802 (0.7288)	1.9058 (0.7016)	0.77
Pause Duration Per Total Speaking Duration	0.1918 (0.1440)	0.2984 (0.2939)	< 0.05*
Pause Duration Per Total Word Count	0.0734 (0.0648)	0.1249 (0.1165)	< 0.05*
Peak Amplitude	0.1583 (0.1068)	0.4051 (0.5661)	< 0.05*
Maximum Peak Amplitude	0.1556 (0.1106)	0.3939 (0.5437)	< 0.05*
Minimum Peak Amplitude	−0.1329 (0.1041)	−0.3463 (0.5182)	< 0.05*
Average Peak Amplitude	0.0065 (0.0029)	0.0239 (0.0301)	< 0.05*

^a^Mean (interquartile range)

*Statistical significance

### Performance evaluation of the prediction model

The performance evaluation of the prediction models for both the training and test sets is presented in Table 5. Among the eight machine learning techniques, RF and LightGBM emerged as the two best-performing classifiers. RF achieved the highest test AUC (0.807), indicating strong overall discrimination ability. However, its low sensitivity (0.275) and F1-score (0.305) suggest limited capability in identifying positive cases, despite its high specificity (0.897). LightGBM on the other hand, demonstrated the most balanced and robust performance across metrics. It achieved a test AUC of 0.784, accuracy of 0.779, and the highest F1-score among all models (0.438). Its balanced sensitivity (0.438) and specificity (0.868) further indicate that LightGBM is effective in distinguishing between MDD and BP cases. XGBoost exhibited moderate performance, with a test AUC of 0.760 and accuracy of 0.736; however, its low precision (0.356) and F1-score (0.386) indicate challenges in handling class imbalance. NB and LR produced slightly lower AUCs (0.671 and 0.699, respectively), with relatively balanced sensitivity and specificity, offering interpretability but weaker overall performance. SVM showed an extreme case, achieving perfect sensitivity (1.000) but zero specificity (0.000), indicating that all instances were classified as positive and thus failing to differentiate between classes. DT and KNN demonstrated the weakest predictive capability, with test AUCs below 0.565 and F1-scores below 0.355, reflecting poor generalizability and limited discriminative power.

**Table 5:** Performance evaluation of the prediction model

Method	Dataset	Metrics					
		AUC	Sensitivity	Specificity	Precision	Accuracy	F1-score
LightGBM	Train	0.812	0.427	0.891	0.528	0.791	0.449
	Test	0.784	0.438	0.868	0.463	0.779	0.438
XGBoost	Train	0.772	0.498	0.810	0.445	0.742	0.449
	Test	0.760	0.425	0.816	0.356	0.736	0.386
RF	Train	0.808	0.278	0.913	0.437	0.775	0.322
	Test	0.807	0.275	0.897	0.397	0.769	0.305
NB	Train	0.699	0.460	0.765	0.350	0.697	0.382
	Test	0.671	0.425	0.752	0.306	0.685	0.352
LR	Train	0.709	0.432	0.813	0.398	0.730	0.394
	Test	0.699	0.388	0.781	0.308	0.700	0.334
SVM	Train	0.731	1.000	0.000	0.217	0.217	0.355
	Test	0.751	1.000	0.000	0.205	0.205	0.340
DT	Train	0.548	0.315	0.781	0.318	0.679	0.292
	Test	0.543	0.313	0.774	0.265	0.679	0.281
KNN	Train	0.520	0.535	0.509	0.229	0.516	0.316
	Test	0.561	0.613	0.523	0.248	0.541	0.352

### Feature importance analysis of random forest and LightGBM

To enhance interpretability and understand which features contributed most strongly to distinguishing BP from MDD, we examined the feature importance outputs of the two best performing classifiers, RF and LightGBM. Feature importance was derived using the Permutation Feature Importance technique implemented in Orange3 (version 3.39.0), which evaluates the contribution of each feature by assessing the decrease in model performance after randomly permuting its values. Both models were trained using the full set of 118 extracted linguistic and acoustic variables.

Across the two classifiers, several features consistently emerged as the most influential predictors. Notably, Average Peak Amplitude, Minimum Peak Amplitude, and Peak Amplitude ranked among the top acoustic variables, suggesting that differences in speech intensity patterns play a central role in distinguishing BP from MDD during euthymic states. In addition to acoustic features, several linguistic indicators showed high predictive value, including Pronouns, Word (spirit), Word (normal), and Word (emotion). These terms may reflect subtle differences in narrative content or self-referential language use between the two diagnostic groups.

RF tended to emphasize acoustic variability indicators, while LightGBM assigned relatively higher importance to specific TF-IDF derived keywords. Despite methodological differences, the two models showed a high degree of convergence in identifying key acoustic parameters as dominant predictors. The consistent prominence of amplitude-related features supports the hypothesis that euthymic BP and MDD patients differ in prosodic patterns, even when overt mood symptoms are minimal.

## Discussion

The main findings in this study revealed that [1] Euthymic MDD and BP groups exhibit comparable emotional outlooks in sentiment analysis; [2] Euthymic MDD patients tend to pause more frequently and for longer durations with greater variability in volume, speech intensity, and generally higher peak amplitude [3] RF algorithm demonstrated the best performance in the test set for AUC (AUC = 0.807) in distinguishing between BP and MDD patients.

Concerning the demographic data (Table 1), BP patients tend to experience higher rates of psychiatric admissions compared to those with MDD. BP is marked by recurrent depressive and manic episodes, both of which contribute to significant morbidity and can require intensive management [47–49]. A nationwide study in Portugal analyzing hospitalizations from 2008 to 2015 reported a total of 20,807 BD-related hospitalizations among 13,300 BD patients with an average rate of 0.196 admissions per year per person [50]. Another study using Truven MarketScan research databases found that during 2014–2015, 34,379 MDD-related hospitalizations among 859,479 MDD patients with an average of 0.02 admissions per year per person [51]. Based on those data, our study population aligns with epidemiological statistics that BP patients tend to have more hospitalizations.

To our knowledge, this is the first study to investigate speech patterns between euthymic BP and MDD patients within a controlled, standardized setting, wherein participants responded to an identical one-minute prompt. Our findings revealed that euthymic BP patients showed a trend of producing more words and exhibited shorter pause durations relative to MDD patients. These differences in verbal output were not accompanied by significant disparities in part-of-speech frequency or sentiment profiles, suggesting that the observed speech differences may not simply reflect linguistic content or emotional valence. Although the underlying mechanisms remain unclear, these findings raise the possibility of residual neurocognitive or affective processing differences persisting during euthymic states in bipolar disorder. Further research is warranted to explore the neural and cognitive correlates contributing to this distinct speech pattern.

A notably flattened and blunted prosodic features, as indicated by lower peak amplitude measures (maximum, minimum and average amplitude), were observed in BP group (*p* < 0.05). This finding is consistent with previous research, which indicates that the pitch variability (measured as pitch standard deviation) was lower in euthymic bipolar patients than in HC [52, 53]. A previous consensus review concludes that movement disorders and broader sensorimotor/psychomotor abnormalities are intrinsic features of numerous psychiatric diseases, rather than merely side effects of antipsychotic medication [54].The underlying mechanism might be related to the emotional blunting and persistent executive dysfunction in BP patients [55–58]. In a recent study, using functional MRI, which found that euthymic bipolar patients exhibit reduced activation in both cortical and subcortical brain regions in response to emotional stimuli compared to HCs [55]. This may reflect persistent deficits in emotional processing and might resulting in a monotone prosody [58]. The adverse effect of psychotropics commonly used in BP may also play a role in excessive psychomotor retardation and slowing of cognition of BP group [59, 60]. However, our study does not include information on patients’ medication use, which needs further investigation in this field.

We aimed to develop models for differentiating BP or MDD patients. In our study, RF was the top performer in terms of AUC (AUC: 0.759) on the test set. This is consistent with previous research findings that the RF is a reliable and valid algorithm, and it is frequently used in studies involving voice analysis [19, 20, 61–65].

### Limitations

In this study, there are several notable limitations. First, a key limitation is the small sample size, particularly the bipolar disorder group (*N* = 41). In the final model, this resulted in only 8 BP patients being available in the testing set, which limits the robustness and generalizability of the model performance. Second, the voice recording time was short, only allowing for a one-minute sample of spoken narratives, which may not fully capture the range of speech characteristics relevant to differentiating between BP and MDD during euthymic states. Third, the linguistic data variables analysis was limited, primarily focusing on ༭andarin-based basic-parts-of-speech and sentiment analysis without exploring more nuanced linguistic features. Fourth, examining patients solely during euthymia does not reflect the common clinical scenario of evaluating individuals presenting with a first episode of depression, in whom distinguishing BP from MDD is particularly challenging. Lastly, the lack of data collection regarding psychotropic medication use and substance use is a limitation. These factors, which can significantly influence speech and linguistic features, could not be corrected for in the current analysis.

## Conclusions

In conclusion, this study demonstrates that speech analysis during euthymic phases holds the potential for distinguishing between BP and MDD. The findings indicate that euthymic BP patients have a higher verbal output, shorter pause durations but with less variability in their speech patterns compared to MDD patients. The RF model showed promising performance in distinguishing between these two groups. However, given the small sample size, the results are preliminary, and replication in larger, independent, and well-characterized cohorts is essential.

## Supplementary Information

Supplementary Material 1

## Data Availability

The datasets generated and analyzed during the current study are not publicly available due to concerns regarding participant privacy and institutional restrictions. However, the data are available from the corresponding author on reasonable request and with approval from the Institutional Review Board of Taichung Veterans General Hospital.

## References

[1] Li C-T, Bai Y-M, Huang Y-L, Chen Y-S, Chen T-J, Cheng J-Y, Su T-P. Association between antidepressant resistance in unipolar depression and subsequent bipolar disorder: cohort study. Br J Psychiatry. 2012;200(1):45–51. 22016435 10.1192/bjp.bp.110.086983

[2] Singh T, Rajput M. Misdiagnosis of bipolar disorder. Psychiatry (Edgmont). 2006;3(10):57–63. 20877548 PMC2945875

[3] Newman SS, Mather VG. ANALYSIS OF SPOKEN LANGUAGE OF PATIENTS WITH AFFECTIVE DISORDERS. Am J Psychiatry. 1938;94:913–42.

[4] Alpert M, Pouget ER, Silva RR. Reflections of depression in acoustic measures of the patient’s speech. J Affect Disord. 2001;66(1):59–69. 11532533 10.1016/s0165-0327(00)00335-9

[5] Renfordt E. Changes of speech activity in depressed patients under pharmacotherapy. Pharmacopsychiatry. 1989;22(S 1):2–4. 2654967 10.1055/s-2007-1014616

[6] Rude S, Gortner E-M, Pennebaker J. Language use of depressed and depression-vulnerable college students. Cogn Emot. 2004;18(8):1121–33.

[7] Yang Y, Fairbairn C, Cohn JF. Detecting depression severity from vocal prosody. IEEE Trans Affect Comput. 2013;4(2):142–50. 26985326 10.1109/T-AFFC.2012.38 PMC4791067

[8] Silva WJ, Lopes L, Galdino MKC, Almeida AA. Voice acoustic parameters as predictors of depression. J Voice. 2024;38(1):77–85. 34353686 10.1016/j.jvoice.2021.06.018

[9] Cummins N, Scherer S, Krajewski J, Schnieder S, Epps J, Quatieri TF. A review of depression and suicide risk assessment using speech analysis. Speech Commun. 2015;71:10–49.

[10] Hashim NW, Wilkes M, Salomon R, Meggs J, France DJ. Evaluation of Voice Acoustics as Predictors of Clinical Depression Scores. J Voice. 2017;31(2):256. 10.1016/j.jvoice.2016.06.006 27473933

[11] Jia Y, Liang Y, Zhu T, editors. An analysis of voice quality of Chinese patients with depression. 2019 22nd Conference of the Oriental COCOSDA International Committee for the Co-ordination and Standardisation of Speech Databases and Assessment Techniques (O-COCOSDA); 2019: IEEE.

[12] Almaghrabi SA, Clark SR, Baumert M. Bio-acoustic features of depression: A review. Biomed Signal Process Control. 2023;85:105020.

[13] Taguchi T, Tachikawa H, Nemoto K, Suzuki M, Nagano T, Tachibana R, et al. Major depressive disorder discrimination using vocal acoustic features. J Affect Disord. 2018;225:214–20. 28841483 10.1016/j.jad.2017.08.038

[14] Shin D, Cho WI, Park CHK, Rhee SJ, Kim MJ, Lee H, et al. Detection of minor and major depression through voice as a biomarker using machine learning. J Clin Med. 2021;10(14):3046. 10.3390/jcm10143046. 34300212 10.3390/jcm10143046 PMC8303477

[15] Menne F, Dörr F, Schräder J, Tröger J, Habel U, König A, et al. The voice of depression: speech features as biomarkers for major depressive disorder. BMC Psychiatry. 2024;24(1):794. 10.1186/s12888-024-06253-6. 39533239 10.1186/s12888-024-06253-6 PMC11559157

[16] Zhao Q, Fan HZ, Li YL, Liu L, Wu YX, Zhao YL, et al. Vocal Acoustic Features as Potential Biomarkers for Identifying/Diagnosing Depression: A Cross-Sectional Study. Front Psychiatry. 2022;13:815678. 35573349 10.3389/fpsyt.2022.815678 PMC9095973

[17] Ettore E, Müller P, Hinze J, Riemenschneider M, Benoit M, Giordana B, et al. Digital Phenotyping for Differential Diagnosis of Major Depressive Episode: Narrative Review. JMIR Ment Health. 2023;10:e37225. 36689265 10.2196/37225 PMC9903183

[18] Kaczmarek-Majer K, Dominiak M, Antosik AZ, Hryniewicz O, Kamińska O, Opara K, et al. Acoustic features from speech as markers of depressive and manic symptoms in bipolar disorder: a prospective study. Acta Psychiatr Scand. 2025;151(3):358-74. 10.1111/acps.13735. 39118422 10.1111/acps.13735 PMC11787917

[19] Lee H-J, Cho C-H, Lee T, Jeong J, Yeom JW, Kim S, et al. Prediction of impending mood episode recurrence using real-time digital phenotypes in major depression and bipolar disorders in South Korea: a prospective nationwide cohort study. Psychol Med. 2023;53(12):5636–44. 36146953 10.1017/S0033291722002847

[20] Faurholt-Jepsen M, Busk J, Frost M, Vinberg M, Christensen EM, Winther O, et al. Voice analysis as an objective state marker in bipolar disorder. Translational Psychiatry. 2016;6(7):e856–e. 27434490 10.1038/tp.2016.123 PMC5545710

[21] Faurholt-Jepsen M, Rohani DA, Busk J, Tønning ML, Vinberg M, Bardram JE, et al. Discriminating between patients with unipolar disorder, bipolar disorder, and healthy control individuals based on voice features collected from naturalistic smartphone calls. Acta Psychiatr Scand. 2022;145(3):255–67. 34923626 10.1111/acps.13391

[22] Antosik-Wójcińska AZ, Dominiak M, Chojnacka M, Kaczmarek-Majer K, Opara KR, Radziszewska W, et al. Smartphone as a monitoring tool for bipolar disorder: a systematic review including data analysis, machine learning algorithms and predictive modelling. Int J Med Inf. 2020;138:104131. 10.1016/j.ijmedinf.2020.104131 32305023

[23] Hamilton M. Development of a rating scale for primary depressive illness. Br J Soc Clin Psychol. 1967;6(4):278–96. 6080235 10.1111/j.2044-8260.1967.tb00530.x

[24] Young RC, Biggs JT, Ziegler VE, Meyer DA. A rating scale for mania: reliability, validity and sensitivity. Br J Psychiatry. 1978;133(5):429–35. 728692 10.1192/bjp.133.5.429

[25] Guy W. ECDEU assessment manual for psychopharmacology. Rockville, MD: US Department of Health, Education, and Welfare, Public Health Service, Alcohol, Drug Abuse, and Mental Health Administration, National Institute of Mental Health; 1976.

[26] Sung SF, Chen CH, Pan RC, Hu YH, Jeng JS. Natural language processing enhances prediction of functional outcome after acute ischemic stroke. J Am Heart Assoc. 2021;10(24):e023486. 34796719 10.1161/JAHA.121.023486 PMC9075227

[27] Aizawa A. An information-theoretic perspective of tf–idf measures. Inf Process Manag. 2003;39(1):45–65.

[28] Ku LW, Ho HW, Chen HH. Opinion mining and relationship discovery using CopeOpi opinion analysis system. J Am Soc Inf Sci Technol. 2009;60(7):1486–503.

[29] Mohammad SM, Turney PD. Nrc emotion lexicon. Natl Res Council Can. 2013;2:234.

[30] Czarnek G, Stillwell D. Two is better than one: using a single emotion lexicon can lead to unreliable conclusions. PLoS One. 2022;17(10):e0275910. 36240202 10.1371/journal.pone.0275910 PMC9565755

[31] De Bruyne L, Atanasova P, Augenstein I. Joint emotion label space modeling for affect lexica. Comput Speech Lang. 2022;71:101257.

[32] Wang Y, Huang G, Li M, Li Y, Zhang X, Li H. Automatically constructing a fine-grained sentiment lexicon for sentiment analysis. Cogn Comput. 2023;15(1):254–71.

[33] Rokach L, Maimon O. Data mining and knowledge discovery handbook. Springer New York; 2010.

[34] Hu Y-H, Hung J-H, Hu L-Y, Huang S-Y, Shen C-C. An analysis of Chinese nursing electronic medical records to predict violence in psychiatric inpatients using text mining and machine learning techniques. PLoS One. 2023;18(6):e0286347. 37285344 10.1371/journal.pone.0286347 PMC10246850

[35] Sung S-F, Hung L-C, Hu Y-H. Developing a stroke alert trigger for clinical decision support at emergency triage using machine learning. Int J Med Inform. 2021;152:104505. 34030088 10.1016/j.ijmedinf.2021.104505

[36] Kramer O. Dimensionality reduction with unsupervised nearest neighbors. Springer; 2013.

[37] Ke G, Meng Q, Finley T, Wang T, Chen W, Ma W, et al. LightGBM: a highly efficient gradient boosting decision tree. Adv Neural Inf Process Syst. 2017;30:3146-54.

[38] Menard S. Applied logistic regression analysis. SAGE publications; 2001.

[39] Steinwart I, Christmann A. Support vector machines. Springer Science & Business Media; 2008.

[40] Friedman N, Geiger D, Goldszmidt M. Bayesian network classifiers. Mach Learn. 1997;29:131–63.

[41] Breiman L. Random forests. Mach Learn. 2001;45(1):5–32.

[42] Chen K, Tsai C-F, Hu Y-H, Hu C-W. The effect of review visibility and diagnosticity on review helpfulness–an accessibility-diagnosticity theory perspective. Decis Support Syst. 2024;178:114145.

[43] Chen T. Xgboost: extreme gradient boosting. R package version 04 – 2. 2015;1(4).

[44] Cheng Q, Li TM, Kwok C-L, Zhu T, Yip PS. Assessing suicide risk and emotional distress in Chinese social media: a text mining and machine learning study. J Med Internet Res. 2017;19(7):e243. 28694239 10.2196/jmir.7276 PMC5525005

[45] Burdisso SG, Errecalde M, Montes-y-Gómez M. A text classification framework for simple and effective early depression detection over social media streams. Expert Syst Appl. 2019;133:182–97.

[46] Tadesse MM, Lin H, Xu B, Yang L. Detection of depression-related posts in reddit social media forum. IEEE Access. 2019;7:44883–93.

[47] Frye MA, Gitlin MJ, Altshuler LL. Unmet needs in bipolar depression. Depress Anxiety. 2004;19(4):199–208. 15274168 10.1002/da.20013

[48] Blader JC, Carlson GA. Increased rates of bipolar disorder diagnoses among US child, adolescent, and adult inpatients, 1996–2004. Biol Psychiatry. 2007;62(2):107–14. 17306773 10.1016/j.biopsych.2006.11.006 PMC2001259

[49] Angst J. The course of major depression, atypical bipolar disorder, and bipolar disorder. In: New results in depression research. Springer; 1986. p. 26–35.

[50] Gonçalves-Pinho M, Freitas A, von Doellinger O, Ribeiro JP. Bipolar Disorder Related Hospitalizations - a Descriptive Nationwide Study Using a Big Data Approach. Psychiatr Q. 2022;93(1):325–33. 34581934 10.1007/s11126-021-09951-6

[51] Citrome L, Jain R, Tung A, Landsman-Blumberg PB, Kramer K, Ali S. Prevalence, treatment patterns, and stay characteristics associated with hospitalizations for major depressive disorder. J Affect Disord. 2019;249:378–84. 30818246 10.1016/j.jad.2019.01.044

[52] Vanello N, Guidi A, Gentili C, Werner S, Bertschy G, Valenza G, et al. Speech analysis for mood state characterization in bipolar patients. Annu Int Conf IEEE Eng Med Biol Soc. 2012;2012:2104–7. 23366336 10.1109/EMBC.2012.6346375

[53] Jakobsen P, Stautland A, Riegler MA, Côté-Allard U, Sepasdar Z, Nordgreen T, et al. Complexity and variability analyses of motor activity distinguish mood states in bipolar disorder. PLoS One. 2022;17(1):e0262232. 35061801 10.1371/journal.pone.0262232 PMC8782466

[54] Walther S, van Harten PN, Waddington JL, Cuesta MJ, Peralta V, Dupin L, et al. Movement disorder and sensorimotor abnormalities in schizophrenia and other psychoses-European consensus on assessment and perspectives. Eur Neuropsychopharmacol. 2020;38:25–39. 32713718 10.1016/j.euroneuro.2020.07.003

[55] Malhi GS, Lagopoulos J, Sachdev PS, Ivanovski B, Shnier R. An emotional Stroop functional MRI study of euthymic bipolar disorder. Bipolar Disord. 2005;7:58–69. 16225562 10.1111/j.1399-5618.2005.00255.x

[56] Mur M, Portella MJ, Martínez-Arán A, Pifarré J, Vieta E. Persistent neuropsychological deficit in euthymic bipolar patients: executive function as a core deficit. J Clin Psychiatry. 2007;68(7):1078–86. 17685745 10.4088/jcp.v68n0715

[57] Torres I, Boudreau V, Yatham L. Neuropsychological functioning in euthymic bipolar disorder: a meta-analysis. Acta Psychiatr Scand. 2007;116:17–26. 17688459 10.1111/j.1600-0447.2007.01055.x

[58] Smith DJ, Muir WJ, Blackwood DH. Neurocognitive impairment in euthymic young adults with bipolar spectrum disorder and recurrent major depressive disorder. Bipolar Disord. 2006;8(1):40–6. 16411979 10.1111/j.1399-5618.2006.00275.x

[59] Nadkarni S, Devinsky O. Psychotropic effects of antiepileptic drugs. Epilepsy Curr. 2005;5(5):176–81. 16175217 10.1111/j.1535-7511.2005.00056.x PMC1201637

[60] Gualtieri CT, Johnson LG. Comparative neurocognitive effects of 5 psychotropic anticonvulsants and lithium. MedGenMed. 2006;8(3):46. 17406176 PMC1781293

[61] Faurholt-Jepsen M, Rohani DA, Busk J, Tønning ML, Winther O, Frost M, et al. Voice analyses using smartphone-based data in patients with bipolar disorder, unaffected relatives and healthy control individuals, and during different affective states. Int J Bipolar Disord. 2021;9(1):28. 10.1186/s40345-021-00243-3. 34850296 10.1186/s40345-021-00243-3 PMC8632566

[62] Wanderley Espinola C, Gomes JC, Mônica Silva Pereira J, dos Santos WP. Detection of major depressive disorder, bipolar disorder, schizophrenia and generalized anxiety disorder using vocal acoustic analysis and machine learning: an exploratory study. Res Biomed Eng. 2022;38(3):813–29.

[63] Perez Arribas I, Goodwin GM, Geddes JR, Lyons T, Saunders KE. A signature-based machine learning model for distinguishing bipolar disorder and borderline personality disorder. Transl Psychiatry. 2018;8(1):274. 30546013 10.1038/s41398-018-0334-0 PMC6293318

[64] Muaremi A, Gravenhorst F, Grünerbl A, Arnrich B, Tröster G, editors. Assessing bipolar episodes using speech cues derived from phone calls. International symposium on pervasive computing paradigms for mental health; 2014: Springer.

[65] Nawas KK, Barik MK, Khan AN, editors. Speaker recognition using random forest. ITM Web of Conferences; 2021: EDP Sciences.

