# Supplementary material for: Speech analysis for differentiating bipolar disorder and major depressive disorder during euthymic states
Source: Ann Gen Psychiatry. 2026 Apr 2;25:39. doi: 10.1186/s12991-026-00652-7 (PMC13169885; doi:10.1186/s12991-026-00652-7)
Supplement: Supplementary file 1 — Supplementary Material 1 [file 12991_2026_652_MOESM1_ESM.docx]

**Supplementary Table 1
Demographic Data of Patients with bipolar disorder (BP) and major depressive disorder (MDD) in Training Group**

| **Variable name** | **BP**  **N= 33 (%)** | **MDD**  **N= 119 (%)** | ***P* values** |
| --- | --- | --- | --- |
| Sex |  |  | 0.99 |
| Male | 9 (27.27) | 32 (26.89) |  |
| Female | 24 (72.73) | 87 (73.11) |  |
| Age (years) ^a^ | 39.94 (16.34) | 41.30 (17.30) | 0.69 |
| Education Level |  |  | 0.56 |
| Elementary school | 0 (0.00) | 5 (4.20) |  |
| Junior high school | 4 (12.12) | 10 (8.40) |  |
| Senior high school | 13 (39.39) | 41 (34.45) |  |
| College degree or higher | 16 (48.48) | 63 (52.94) |  |
| Occupation | 18 (54.55) | 57 (47.90) | 0.63 |
| Marriage |  |  | 0.26 |
| Unmarried | 17 (51.52) | 60 (50.42) |  |
| Married | 11 (33.33) | 51 (42.86) |  |
| Divorced | 5 (15.15) | 8 (6.72) |  |
| Suicide History | 14 (42.42) | 40 (33.61) | 0.47 |
| Psychiatric Hospitalization History |  |  | <0.05* |
| No psychiatric hospitalization history | 18 (54.55) | 92 (77.31) |  |
| Hospitalized within the past year | 1 (3.03) | 12 (10.08) |  |
| Hospitalized more than one year ago | 14 (42.42) | 15 (12.61) |  |
| Residential Status |  |  | 0.99 |
| Living alone | 6 (18.18) | 21 (17.65) |  |
| Living with family or friends | 27 (81.82) | 98 (82.35) |  |
| Alcohol Consumption Pattern |  |  | 0.36 |
| Less than three times per week | 30 (90.91) | 115 (96.64) |  |
| Three times per week and above | 3 (9.09) | 4 (3.36) |  |
| Number of Physical Illnesses | 0.67 (0.85) | 0.40 (0.73) | 0.08 |

*Statistical significance

**Supplementary Table 2
Demographic Data of Patients with bipolar disorder (BP) and major depressive disorder (MDD) in Testing Group**

| **Variable name** | **BP**  **N= 8 (%)** | **MDD**  **N= 31 (%)** | ***P* values** |
| --- | --- | --- | --- |
| Sex |  |  | 0.54 |
| Male | 3 (37.50) | 6 (19.35) |  |
| Female | 5 (62.50) | 25 (80.65) |  |
| Age (years) ^a^ | 39.75 (9.68) | 47.03 (17.26) | 0.26 |
| Education Level |  |  | 0.71 |
| Elementary school | 0 (0.00) | 4 (12.90) |  |
| Junior high school | 1 (12.50) | 3 (9.68) |  |
| Senior high school | 2 (25.00) | 9 (29.03) |  |
| College degree or higher | 5 (62.50) | 15 (48.39) |  |
| Occupation | 6 (75.00) | 16 (51.61) | 0.43 |
| Marriage |  |  | 0.43 |
| Unmarried | 5 (62.50) | 13 (41.94) |  |
| Married | 3 (37.50) | 14 (45.16) |  |
| Divorced | 0 (0.00) | 4 (12.90) |  |
| Suicide History | 3 (37.50) | 12 (38.71) | 0.99 |
| Psychiatric Hospitalization History |  |  | <0.05* |
| No psychiatric hospitalization history | 2 (25.00) | 20 (64.52) |  |
| Hospitalized within the past year | 1 (12.50) | 5 (16.13) |  |
| Hospitalized more than one year ago | 5 (62.50) | 6 (19.35) |  |
| Residential Status |  |  | 0.16 |
| Living alone | 0 (0.00) | 10 (32.26) |  |
| Living with family or friends | 8 (100.00) | 21 (67.74) |  |
| Alcohol Consumption Pattern |  |  | 0.99 |
| Less than three times per week | 8 (100.00) | 29 (93.55) |  |
| Three times per week and above | 0 (0.00) | 2 (6.45) |  |
| Number of Physical Illnesses | 0.75 (1.04) | 0.52 (0.77) | 0.48 |

*Statistical significance
